# Genomic characterization of infectious bronchitis viruses isolated from retail poultry meat in South Korea, 2017–2023

**DOI:** 10.3389/fvets.2025.1735504

**Published:** 2026-01-12

**Authors:** Heesu Lee, Sun-Hak Lee, Jun-Young Kim, Dam-Hee Park, Andrew Y. Cho, Deok-Hwan Kim, Tae-Hyeon Kim, Seo Jeong An, Juho Song, Yun-Jeong Choi, Ye-Ram Seo, Dong-Yeop Lee, Ji-Yeon Hyeon, Dong-Hun Lee, Chang-Seon Song

**Affiliations:** 1 Avian Disease Laboratory, College of Veterinary Medicine, Konkuk University, Seoul, Republic of Korea; 2 Wildlife Health Laboratory, College of Veterinary Medicine, Konkuk University, Seoul, Republic of Korea

**Keywords:** GI-15 lineage, GI-19 lineage, infectious bronchitis virus, phylogenetic analysis, retail poultry meat, surveillance

## 1 Introduction

Infectious bronchitis virus (IBV), first identified in the 1930s, continues to impose a significant economic burden on the global poultry industry due to its capacity to cause acute and highly contagious respiratory, renal, and reproductive diseases in chickens (1). This virus, classified as a positive-sense single-stranded RNA virus within the family *Coronaviridae* and the genus *Gammacoronavirus*, exhibits considerable genetic variability, which complicates efforts toward effective disease control (2). The viral spike (S) glycoprotein, which mediates host cell entry, is composed of two subunits, S1 and S2. While the S1 subunit is responsible for receptor binding, tissue tropism, and serotype specificity, the S2 subunit functions to anchor the spike protein to the viral envelope, facilitating viral fusion and entry (3, 4). Among these, the S1 coding sequence is particularly prone to mutation, and its high variability has been identified as a major factor contributing to immune evasion and vaccine failure, thereby making it a primary target for molecular classification and phylogenetic analysis of IBV strains (2). The evolution of IBV is driven by both antigenic drift and genetic recombination, which enable the emergence of new variants with distinct antigenic properties (5). Such genetic characteristics can compromise the efficacy of existing vaccines, underscoring the necessity for continuous surveillance to monitor circulating strains and evaluate their genetic characteristics.

In South Korea, four genotypes have been reported to date—GI-15, GI-16, GI-19, and GVI-1 (6). IBV was first documented in Korea in 1986, initially designated as Korean Group I (K-I) following the respiratory disease outbreaks in poultry (7). This group was reclassified under the GI-15 lineage (8). Subsequently, in 1990, a nephropathogenic variant, Korean Group II (K-II), specifically the KM91-like strain, emerged and caused severe economic losses (9). Around 2003 to 2006, the QX genotype—originally detected in China in 1996 (10) and known for its international spread (11)—was introduced into Korea and has since been referred to as the QX-like and KM-91-like variant within the K-II subgroup (12, 13). In 2009, a recombinant strain combining features of the KM91-like and QX-like lineages, known as K40/09-like, was identified and has become one of the prevalent strains in Korea (13, 14). These findings reflect a complex pattern of IBV evolution in the region, with multiple recombination events contributing to the observed genetic diversity. Recent studies have further revealed the presence of at least four phylogenetically distinct QX-like subgroups in Korea, which differ from previously reported lineages and suggest multiple independent introductions of the virus (13). A phylogeographic analysis of QX-like viruses indicated that these variants were introduced into Korea on at least four separate occasions between 2001 and 2020 (13). Considering the continued genetic evolution and the repeated emergence of novel variants, comprehensive and sustained molecular surveillance is essential to ensure the early detection and effective control of IBV in poultry populations.

Current surveillance in Korea largely targets clinically affected flocks on farms, which can introduce sampling bias and miss subclinical circulation beyond production sites. Here, we utilized retail poultry meat as a post-farm sentinel with following objectives: (1) to detect IBV genotypes that persist through slaughter and processing; (2) to capture strains not targeted by clinical farm sampling, including those from asymptomatic flocks aggregated via shared slaughter and distribution; and (3) to compare lineage composition with farm-origin and vaccine strains to identify potential gaps in farm-based surveillance. Because retail products pool birds from multiple farms and batches, retail sampling provides a system-level snapshot of regional circulation and mixed-source contamination that may be overlooked by single-farm investigations. Accordingly, we conducted molecular detection and S1-based genotyping of IBV from retail meat collected nationwide in South Korea (2017–2023) to determine the added value of this complementary, post-farm surveillance approach.

## 2 Materials and methods

### 2.1 Sample collection and virus isolation

**Figure 1:**
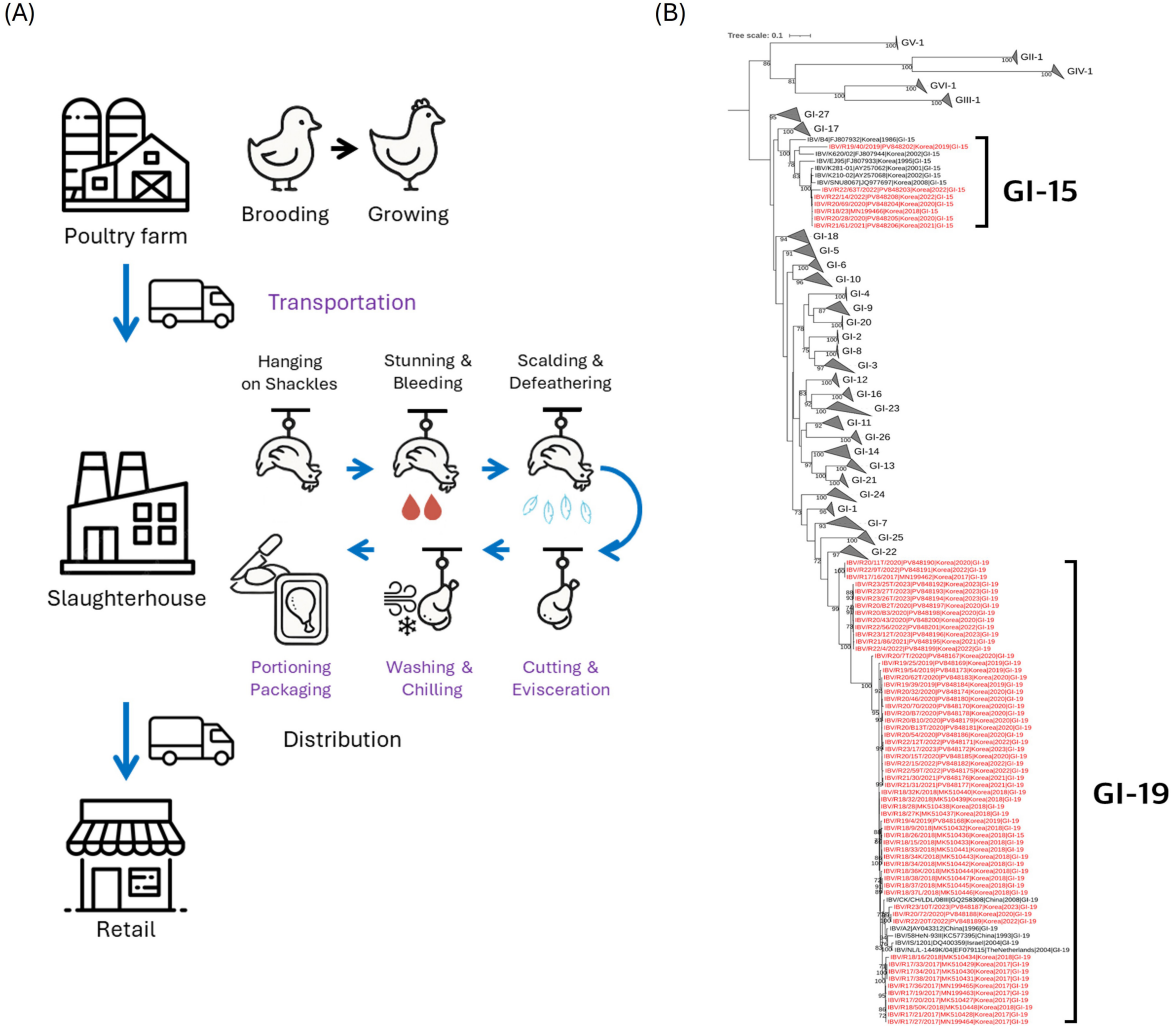
Schematic overview of the retail poultry meat production process and phylogenetic relationships of infectious bronchitis viruses (IBVs) detected in retail meat samples collected in South Korea from 2017 to 2023. **(A)** Cold-chain production process illustrating potential viral contamination points (purple), indicating stages where virus exposure or transfer may occur through surface contact, shared equipment, or water systems. **(B)** Maximum-likelihood phylogenetic tree of IBVs based on the S1 coding sequence, constructed in RAxML using the Tamura–Nei model with 1,000 bootstrap replicates. All lineages except GI-15 and GI-19 were compressed for clarity. Korean IBVs isolated from retail poultry meat are shown in red text.

Retail poultry products were purchased through online delivery services in South Korea during 2017–2023. Detailed information on the type, location, and sampling date of each retail poultry meat sample is provided in Supplementary Table 1. Each sample was a whole carcass, with product weights ranging from 500 to 1,600 g. Products were transported under cold-chain conditions in insulated boxes with ice packs, delivered within 2 days of ordering, and processed for testing on the day of receipt. Considering the potential for viral contamination during multiple stages of the cold-chain production process—including transportation, cutting, evisceration, chilling, portioning, and packaging, as illustrated in Figure 1A—we processed retail poultry meat following procedures below.

Retail poultry meat was separately placed in a sterile plastic bag containing 300 ml of phosphate-buffered saline (PBS) and manually agitated for 2 min. The homogenate rinse was collected and clarified using centrifugation at 3,000 rpm for 10 min. Additionally, leftovers of tissues such as kidneys and lungs from retailed meat samples were collected under sterile conditions and processed by homogenization and clarified by centrifugation at 3,000 rpm for 10 min. Virus isolation was performed by inoculating 0.2 ml of the processed supernatant into the allantoic cavity of 9- to 11-day-old specific-pathogen-free (SPF) embryonated chicken eggs. Eggs were incubated at 37 °C for 72 h, and allantoic fluids were harvested after two blind passages. The allantoic fluid harvested from the eggs was tested for hemagglutination (HA) assay to screen viruses including avian influenza virus (AIV) and Newcastle disease virus (NDV) (15).

### 2.2 Viral RNA extraction, RT-PCR, and sequencing

Viral RNA was extracted from harvested allantoic fluid using the RNeasy kit (QIAGEN, Hilden, Germany). Detection of IBV was performed by real-time quantitative reverse transcription polymerase chain reactions (qRT-PCR) using Qiagen Quantitect RT-PCR reagents (Qiagen, Manchester, UK), following a previously described protocol (16). For qRT-PCR-positive samples, complementary DNA (cDNA) was synthesized using SuperScript IV reverse Transcriptase (Life Technologies) and random primers according to the manufacturer's instructions. Samples for sequencing were selected based on the collection region and sampling date to ensure a representative geographic and temporal distribution; when multiple products from the same region and company were available, a single representative sample was sequenced to avoid redundancy. The complete S1 coding sequence was amplified using two primer sets described in a previous study (6). PCR products were purified with the GeneJET Gel Extraction Kit (Thermo Fisher Scientific, Waltham, MA, USA) and sequenced by Sanger sequencing (Macrogen Co., Ltd., Seoul, South Korea).

For HA positive samples, qRT-PCR assays targeting the matrix (M) gene of AIV (17) and NDV (18) were conducted. NDV-positive samples were further analyzed by amplifying and Sanger sequencing the fusion (F) gene to determine lineage and pathotype (19).

### 2.3 Phylogenetic analysis

To determine the global lineage based on the S1 phylogeny-based classification system (8), S1 coding sequences, including representative sequences for each genotype, were retrieved from the GenBank database. To further investigate the genetic relationships with global IBV lineages, additional S1 sequences corresponding to the identified lineages, along with sequences showing high nucleotide identity in BLASTn searches, were also obtained from GenBank. For each sequence, the top 100 hits with the highest nucleotide identity were selected. Sequences showing 99% nucleotide identity were removed using the ElimDupes tool to eliminate redundancy (20). All sequences were aligned using MAFFT v7.308 for multiple sequence alignment (21). A maximum likelihood (ML) phylogenetic tree was constructed using RAxML with the general time-reversible (GTR) nucleotide substitution model and gamma distribution, employing 1,000 rapid bootstrap replicates (22). The resulting maximum likelihood tree was visualized using iTOL (Interactive Tree of Life, v6) (https://itol.embl.de/). To identify cluster-specific amino-acid substitutions within GI-19, we compared S1 hypervariable region (HVR) I–III amino-acid sequences by cluster.

## 3 Descriptive results

Between 2017 and 2023, 511 retail poultry meat samples—including broiler chicken, Korean native chicken (NC), and broiler duck—were collected from commercial markets and delivery services in South Korea (Supplementary Table 1). Eighty-nine samples tested positive for IBV from homogenate rinse fluid, yielding an overall detection rate of 17.4%, while 57 out of 461 leftover tissues were positive (12.4%) (Supplementary Table 2). Annual positivity ranged from 44.0% in 2018 to 5.5% in 2023, and IBV was detected across multiple provinces, with higher rates in Chungcheong-do (29.5%) and Jeolla-do (24.7%) (Supplementary Table 3). Monthly prevalence peaked in April (47.3%) and declined during the summer months, suggesting seasonal variation with higher circulation in spring—patterns consistent with earlier reports of enhanced IBV activity during cooler periods (Supplementary Table 4). These results indicate that IBV can persist post-slaughter, emphasizing the potential of retail meat as a matrix for detecting subclinical infections circulating in commercial flocks. No AIV was detected by hemagglutination assay or qRT-PCR, whereas NDV was confirmed in 74 of 511 samples (14.4%); sequencing of the F gene revealed that all NDV isolates corresponded to commercial vaccine strains, Avinew^®^ (Supplementary Table 1).

Of the 122 qRT-PCR–positive samples, 67 IBV isolates were selected for S1-coding sequence analysis based on collection region and sampling date to ensure representative geographic and temporal coverage, while isolates derived from identical brands or collected on the same date within the same period were excluded to avoid redundancy (Supplementary Table 5). Phylogenetic analysis on S1 coding sequences identified two major lineages, GI-15 (*n* = 7) and GI-19 (*n* = 60), both previously reported in South Korean poultry (Figure 1B). In South Korea, IBV circulation has long been dominated by GI-19 (QX-like/K-IId) with GI-15 persisting, alongside historically reported GI-16 and GVI-1 (6, 13, 23); the retail-derived lineages observed here are therefore consistent with the national genotype landscape.

**Figure 2:**
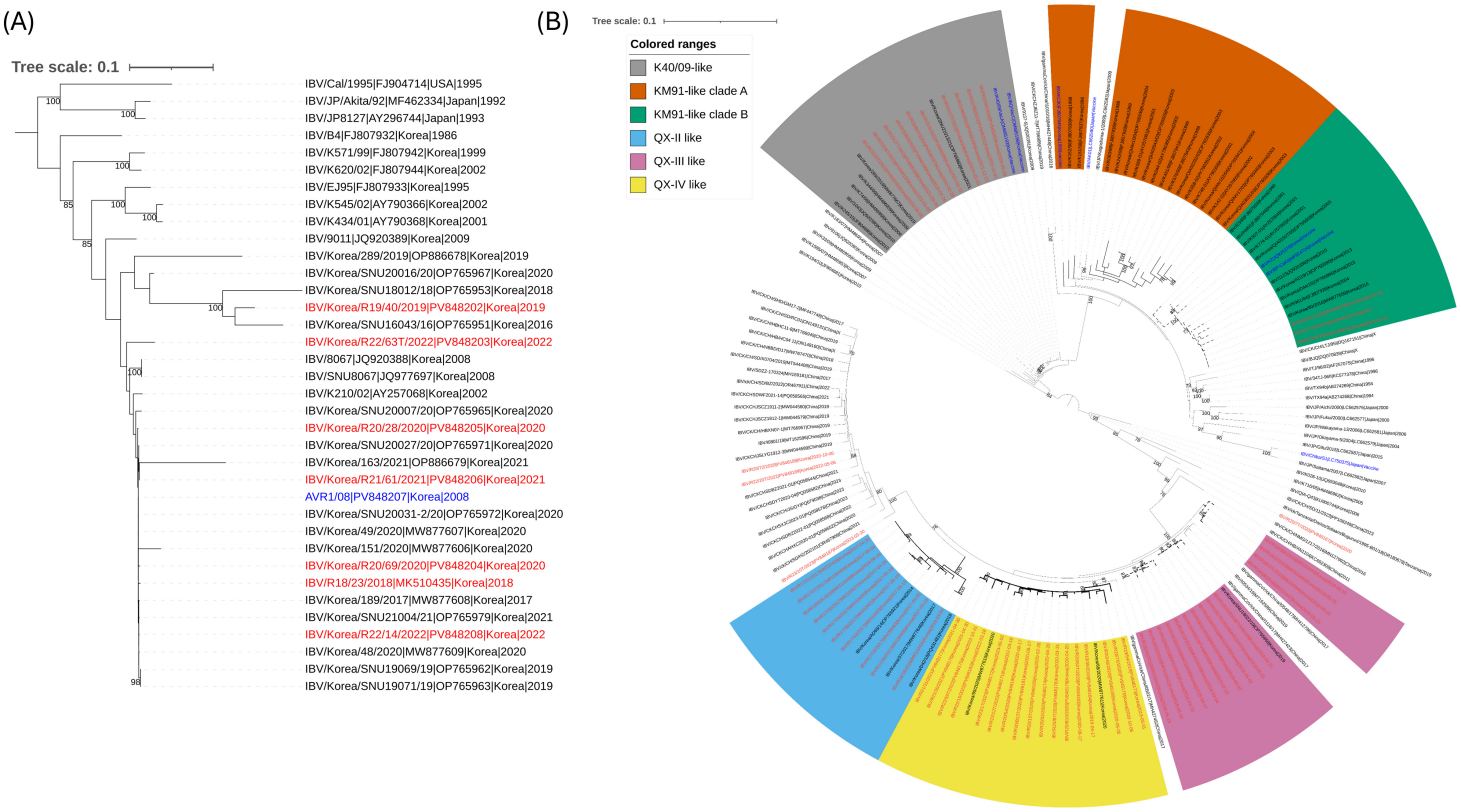
Phylogenetic analysis of infectious bronchitis viruses (IBVs) isolated from retail poultry meat samples in South Korea from 2017 to 2023 based on the full-length S1 coding sequence. **(A)** GI-15 lineage IBVs. **(B)** GI-19 lineage IBVs. Both trees were constructed using the maximum-likelihood method in RAxML with the Tamura–Nei substitution model and 1,000 bootstrap replicates. Korean IBVs isolated from retail poultry meat are shown in red text, and vaccine strains are indicated in blue text.

GI-15 isolates, all derived from broiler chicken meat except one KNC, shared 85.6–99.7% nucleotide identity and clustered with respiratory-type field strains described in prior outbreaks (7, 9, 13, 24, 25) (Figure 2A). Phylogenetic analysis of GI-19 lineage isolates showed clustering within subgroups related to Korean vaccine strains (KM91-, K40/09-like), as well as QX-II, QX-III, and QX-IV types, collectively referred to as the K-IId subgenotype (13, 23) (Figure 2B). Most QX-IV isolates originated from broilers, whereas QX-II and QX-III types were less frequent, suggesting temporal shifts among sub-lineages. Several isolates exhibited >99% identity with commercial vaccine strains such as K40/09 or K2, indicating possible vaccine origin or persistence of vaccine-derived viruses in processing environments. Conversely, a few isolates (e.g., R20/7T/2020, R22/20T/2022, R23/10T/2023) clustered with Chinese strains, implying occasional introductions or independent evolution. Overall, retail-derived isolates showed close genetic similarity to contemporary farm-origin strains, particularly within the GI-19 lineage. Across the S1 hypervariable regions (HVR), we detected substitutions at multiple sites as summarized in Supplementary Table 6. A subset of sites was cluster-informative, segregating Gi-19 subgroups, supporting the phylogenetic lineage assignments. Notably, multiple amino-acid substitutions were detected across regions overlapping HVRs—including approximately aa 24–61, 132–149, and 291–398—previously reported in neutralization-resistant variants (26). We also noted patterns consistent with the presence of mosaic substitution constellations within the sialic-acid binding domain (S1 N-terminal aa 19–227), a region linked to virulence, nephrotropism, and serotype switching (27). While these patterns indicate potential phenotypic relevance, definitive functional effects cannot be inferred from S1 variation alone; future work integrating full-genome data, glycosylation motif mapping (N-X-S/T), receptor-binding assays, and cross-neutralization studies will be required to determine whether specific substitutions among these sites modulate tropism or vaccine match.

This study was designed to evaluate retail poultry meat as a post-farm sentinel with three objectives: (i) detect IBV genotypes that persist through slaughter/processing (supported here by recovery in embryonated eggs), (ii) capture strains not targeted by clinical farm sampling because retail products pool asymptomatic flocks across shared slaughter/distribution, and (iii) compare lineage composition with farm and vaccine strains to identify gaps in farm-based surveillance. We acknowledge that farm investigations are more informative for source attribution and transmission, and that retail detections can reflect slaughter-surviving or environmentally stable virus and, at times, processing-related surface contamination; foodborne risk was not assessed. Within this framework, the concurrent recovery of GI-15 and GI-19 from retail products—along with vaccine-like and field-like sequences—indicates that the genotypes circulating in production systems are also detectable post-farm, providing a system-level snapshot of regional circulation and mixed-source contamination that single-farm studies may miss. These genomic findings support the epidemiologic value of retail sampling as a complementary tool to monitor lineage diversity, track potential vaccine spillover/persistence, and inform targeted vaccine selection. While our analyses were limited to the S1 gene, integrating retail-based surveillance with farm studies and full-genome/phenotypic data will better resolve contamination vs. true persistence and strengthen IBV control strategies.

## Data Availability

The datasets presented in this study can be found in online repositories. The names of the repository/repositories and accession number(s) can be found in the article/Supplementary material.

## Supplementary material

Supplementary Table 1 Sample collection information including year, region, poultry type, sample type (tissue homogenate or rinse water), and the number of samples tested for IBV in South Korea, 2017–2023.

Supplementary Table 2 Detection rates of infectious bronchitis virus (IBV) by retail poultry meat type and sample type, 2017–2023.

Supplementary Table 3 Detection rates of infectious bronchitis virus (IBV) by province of sample production, 2017–2023.

Supplementary Table 4 Monthly detection rates of infectious bronchitis virus (IBV) among retail poultry meat samples collected in South Korea from 2017 to 2023.

Supplementary Table 5 Information on IBV isolates subjected to sequencing, including isolate name, collection date, host type, region, and GenBank accession number.

Supplementary Table 6 Amino-acid substitutions in S1 hypervariable regions (HVR I–III) detected in infectious bronchitis virus (IBV) GI-19 lineages from Korean retail poultry meats, 2017–2023.

Supplementary Data Sheet 1. Phylogenetic tree of infectious bronchitis viruses (IBVs) isolated from retail poultry meat samples in South Korea from 2017 to 2023 based on the S1 coding sequence. The tree was created by the maximum-likelihood method in RAxML using Tamura-Nei model and 1,000 bootstrap replicates. Korean IBVs isolated from retail poultry meat are indicated in red text.
