# Supplementary figures and images for: Genomic characterization of infectious bronchitis viruses isolated from retail poultry meat in South Korea, 2017–2023
Source: Front Vet Sci. 2026 Jan 12;12:1735504. doi: 10.3389/fvets.2025.1735504 (PMC12833514; doi:10.3389/fvets.2025.1735504)

Tree scale: 0.1

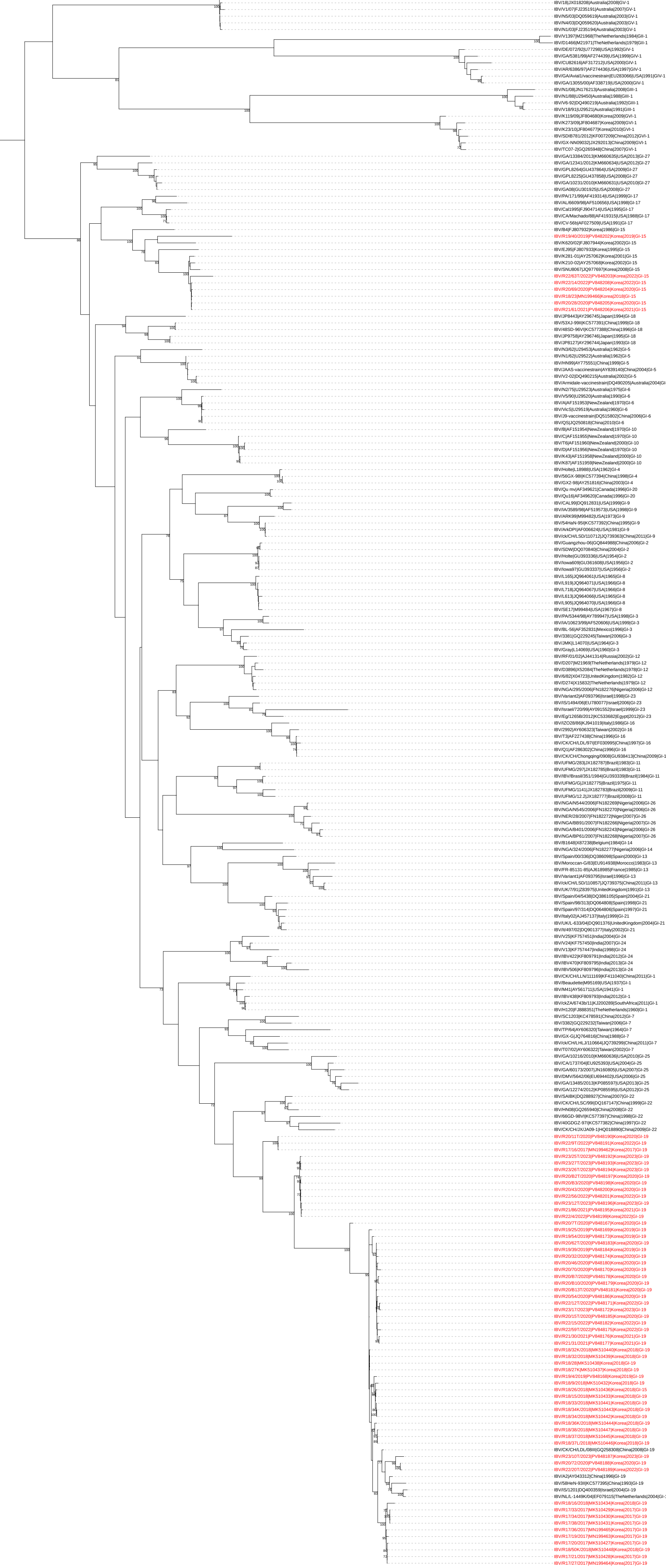

Supplement: Supplementary Data Sheet 1. — Phylogenetic tree of infectious bronchitis viruses (IBVs) isolated from retail poultry meat samples in South Korea from 2017 to 2023 based on the S1 coding sequence. The tree was created by the maximum-likelihood method in RAxML using Tamura-Nei model and 1,000 bootstrap replicates. Korean IBVs isolated from retail poultry meat are indicated in red text. [file Data_Sheet_1.pdf]
